# The Effects of Iodine Attenuation on Pulmonary Nodule Volumetry using Novel Dual-Layer Computed Tomography Reconstructions

**DOI:** 10.1007/s00330-017-4938-1

**Published:** 2017-07-04

**Authors:** A. M. den Harder, F. Bangert, R. W. van Hamersvelt, T. Leiner, Julien Milles, A. M. R. Schilham, M. J. Willemink, P. A. de Jong

**Affiliations:** 1 Department of Radiology, University Medical Center Utrecht, P.O. Box 85500, E01.132, 3508 GA Utrecht, The Netherlands; 2 Department of Radiology, Sint Antonius Ziekenhuis, P.O. Box 2500, 3430EM Nieuwegein, The Netherlands; 3 Philips Healthcare, Best, The Netherlands

**Keywords:** Computed Tomography, Iodine, Dual–Energy, Multiple Pulmonary Nodules, Volume CT

## Abstract

**Objectives:**

To assess the effect of iodine attenuation on pulmonary nodule volumetry using virtual non-contrast (VNC) and mono-energetic reconstructions.

**Methods:**

A consecutive series of patients who underwent a contrast-enhanced chest CT scan were included. Images were acquired on a novel dual-layer spectral CT system. Conventional reconstructions as well as VNC and mono-energetic images at different keV levels were used for nodule volumetry.

**Results:**

Twenty-four patients with a total of 63 nodules were included. Conventional reconstructions showed a median (interquartile range) volume and diameter of 174 (87 – 253) mm^3^ and 6.9 (5.4 – 9.9) mm, respectively. VNC reconstructions resulted in a significant volume reduction of 5.5% (2.6 – 11.2%; p<0.001). Mono-energetic reconstructions showed a correlation between nodule attenuation and nodule volume (Spearman correlation 0.77, (0.49 – 0.94)). Lowering the keV resulted in increased volumes while higher keV levels resulted in decreased pulmonary nodule volumes compared to conventional CT.

**Conclusions:**

Novel dual-layer spectral CT offers the possibility to reconstruct VNC and mono-energetic images. Those reconstructions show that higher pulmonary nodule attenuation results in larger nodule volumes. This may explain the reported underestimation in nodule volume on non-contrast enhanced compared to contrast-enhanced acquisitions.

***Key Points*:**

• *Pulmonary nodule volumes were measured on virtual non-contrast and mono-energetic reconstructions*

• *Mono-energetic reconstructions showed that higher attenuation results in larger volumes*

• *This may explain the reported nodule volume underestimation on non-contrast enhanced CT*

• *Mostly metastatic pulmonary nodules were evaluated, results might differ for benign nodules*

## Introduction

Pulmonary nodules are a frequent finding on chest CT examinations and are expected to become even more frequent due to the recent recommendations regarding lung cancer screening [1, 2]. Most pulmonary nodules do not require immediate action and are followed over time to assess change in size. This is most accurately assessed by volumetry, which is also recommended as the preferred method in the recent guidelines of the British Thoracic Society [3]. A volume change of ≥25% is considered a significant growth. Volume measurements can be affected by several parameters, including slice thickness, reconstruction method, volumetry software package, and the use of contrast media [4–9].

In the past decade dual-energy CT systems became available which offer new reconstruction options. Conventional CT uses one X-ray tube and a single-layer detector. However, tissues with different compositions can exhibit the same attenuation and, therefore, result in similar Hounsfield Units (HU). Dual-energy CT separates low- and high-energy X-ray photons, which provides additional information resulting in improved tissue contrast [10]. Currently, different approaches for dual-energy are available including the use of two X-ray tubes at different voltages, rapid switching of the tube potential during a gantry rotation, switching of the tube potential between gantry rotations or a dual-layer detector, which separates low- and high-energy photons [10, 11].

With dual-energy CT, iodine voxels can be identified, which allows for the removal of iodine, resulting in a virtual non-contrast (VNC) reconstruction. Also, virtual mono-energetic images can be reconstructed for a spectrum of energy levels (40-200 keV) [12]. Due to the high k-edge of iodine at 33.2 keV, iodine attenuation is boosted more at low keV levels than other materials. This allows for assessment of the effect of iodine attenuation on pulmonary nodule volumetry without the influence of inter-scan variation. To the best of our knowledge, the effect of VNC and mono-energetic images reconstructed by novel dual-layer spectral CT on pulmonary nodule volume has not been evaluated yet. Therefore, the aim of our study was to assess the effect of iodine attenuation on pulmonary nodule volumetry using VNC and virtual mono-energetic reconstructions.

## Methods

### Study population

This study was part of a prospective study on the clinical value of a novel dual-layer spectral CT system and was approved by the local institutional review board at the University Medical Center Utrecht in the Netherlands (number 15/548). All patients signed broad informed consent to use the spectral data for research purposes. A consecutive series of 24 patients who underwent a contrast-enhanced chest CT and had one or more non-calcified solid nodules were included from February until May 2016.

### CT acquisition

All acquisitions were performed on an IQon spectral CT system (Philips Healthcare, Best, The Netherlands). This system uses a single X-ray tube and a dual-layer detector, which separates low (upper detector layer) and high energy photons (bottom detector layer). By combining the information of both layers, a conventional image can be reconstructed. Therefore, dual-energy results are always available for each acquisition with this CT system. A spiral mode with a tube voltage of 120 kVp was used with automatic dose modulation. Acquisition parameters were: collimation 64x0.625 mm, pitch 1.171 and a gantry rotation time of 0.33 seconds. Conventional images, VNC and virtual mono-energetic reconstructions were made at a slice thickness of 1.0 mm and increment of 0.7 mm using vendor recommended settings including spectral level 3 and kernel C. Spectral is a model based iterative reconstruction, developed for dual energy reconstructions. Spectral levels range from 1 to 5, higher levels result in less noise and vice versa. Mono-energetic images were reconstructed at 40, 70, 100, 130, 160 and 200 keV, respectively. This includes the minimum (40 keV) and maximum (200 keV) level as well as the level which is most comparable to the 120 kVp conventional reconstruction (70 keV) [13].The dose length product (DLP) of each scan was recorded. The size-specific dose estimate (SSDE) of the chest was calculated by multiplying the volumetric CT dose index (CTDIvol) with the size specific conversion factor [14]. The CTDIvol at the level of the bifurcation of the pulmonary artery was derived from the DICOM file. The anterior-posterior diameter and lateral diameter were measured on the same level using electronic callipers.

### Volumetry

One observer (FB) with 4 years of experience in chest imaging assessed the CT scans for pulmonary nodules. Only non-calcified solid nodules (more or less round solid lesions smaller than 30 mm in diameter) were included with a maximum of six nodules per patient. A single observer (AH) measured the volume of the nodules using semi-automatic software (IntelliSpace version 8, Philips Healthcare, Best, The Netherlands). After clicking on a nodule, the software algorithm quantified the volume, diameter and mean attenuation of the nodule.

### Sample size

Since prior information about the effect of virtual non-contrast and mono-energetic reconstructions on pulmonary nodule volumetry is lacking, a sample size could not be calculated adequately. Several previous studies investigated the effect of contrast material on pulmonary nodule volumetry and included a median number of 60 nodules [4, 5, 9, 15, 16]. Therefore, at least 60 nodules should be included to allow for a comparison with those studies.

### Statistical analysis

Statistical analysis was performed using SPSS version 21 for Windows (IBM Corp. New York, United States). The nodule volumes measured on VNC and mono-energetic reconstructions were compared to the conventional reconstruction using a Friedman test. Post-hoc analyses were performed using Wilcoxon signed rank tests. A Bonferroni corrected p-value of 0.007 (0.05 divided by seven comparisons) was used for post-hoc analyses. Differences were also visually analysed using Bland-Altman plots. Overall analyses as well as separate analyses for small (≤200 mm^3^) and large (>200 mm^3^) nodules were performed. Correlations between nodule volume and attenuation at different virtual mono-energetic reconstructions were assessed per nodule using the Spearman rank correlation. The correlation was interpreted as very weak (0.00 – 0.19), weak (0.20 – 0.39), moderate (0.40 – 0.59), strong (0.60 – 0.79) or excellent (0.80 – 1.00). Values are provided as median (interquartiles, IQR) unless stated otherwise.

## Results

Twenty-four patients with a total of 63 nodules were included. Median age was 64 (53 – 67) years. Sixty-three percent (15/24) was male. Most patients (79%, *n*=19) underwent a combined CT scan of chest and abdominopelvicregion (DLP 465 (269 – 661) mGy*cm) while five patients (21%) received only a chest CT scan (DLP 74 (68 – 133) mGy*cm). The SSDE of the chest was 3.5 (2.9 – 4.7) mGy.

Clinical diagnoses included metastasis of melanoma (*n*=4), colorectal carcinoma (*n*=5), head and neck carcinoma (*n*=3), oesophageal carcinoma (*n*=2), uterus carcinoma (*n*=2), primary lung carcinoma (*n*=1), ovarian carcinoma (*n*=1), urothelial carcinoma (*n*=1), hemangiopericytoma (*n*=1), cholangiocarcinoma (*n*=1), endometrium carcinoma (*n*=1), seminoma (*n*=1) and an incidentally found pulmonary nodule (*n*=1). The median volume of the nodules was 174 (87 – 523) mm^3^. The median diameter was 6.9 (5.4 – 9.9) mm. Thirty-eight nodules (60.3%) were ≤200 mm^3^ and 25 nodules (39.7%) were >200 mm^3^.

**Fig. 1:**
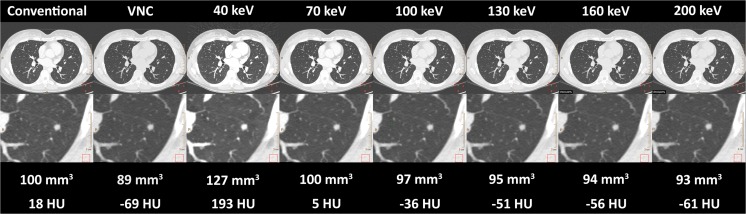
Image appearance at different reconstructions in a 30-year-old female with colorectal carcinoma. Below each image the volume (mm3) and HU-value of the nodule is provided. *VNC virtual non-contrast, keV kiloelectron Volt*

**Fig. 2:**
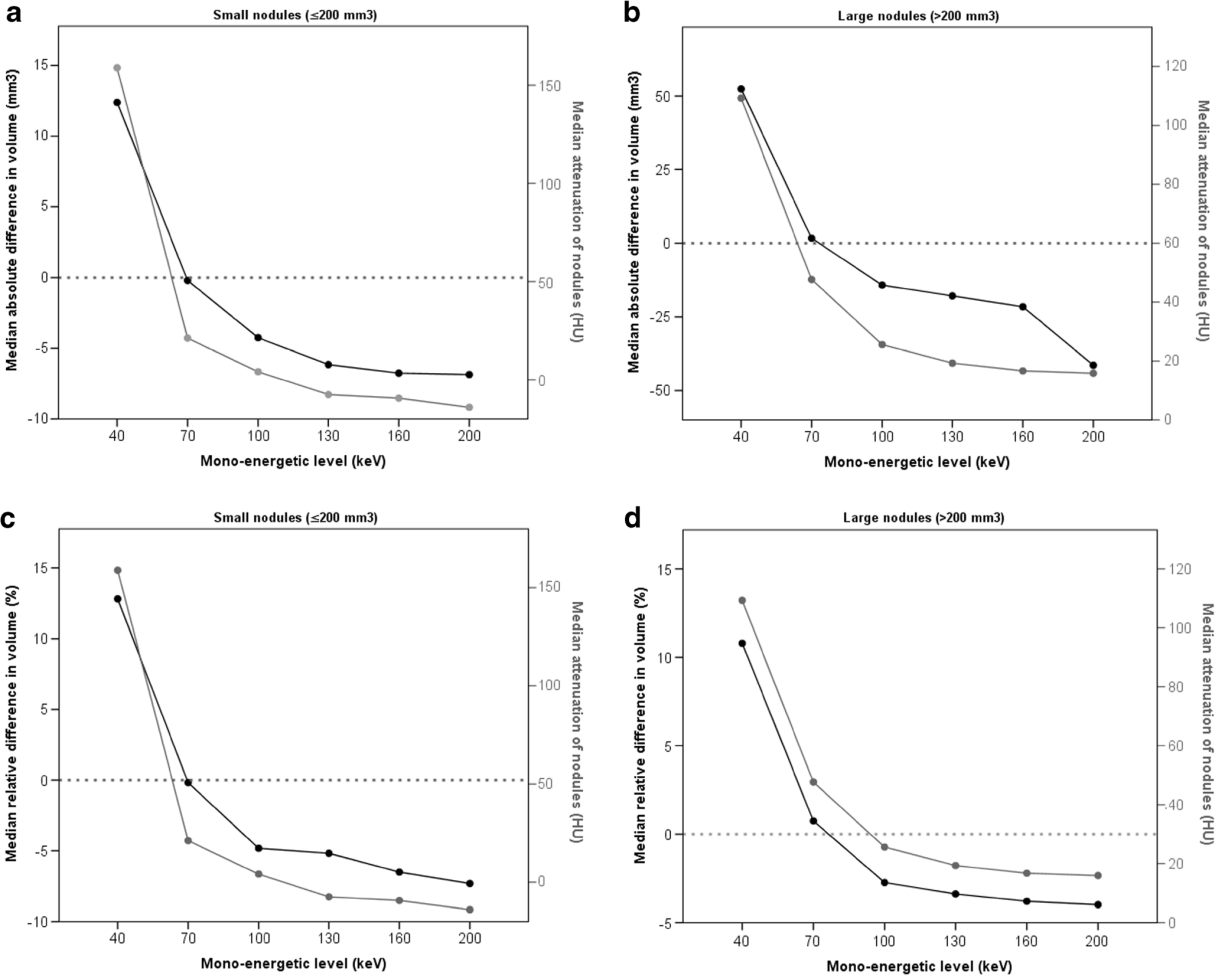
Relationship between nodule volumes measured at different keV-levels and average attenuation of the nodules. The upper two figures (A-B) show absolute differences in volume (compared to conventional) for the different mono-energetic levels while the lower two figures (C-D) represent relative differences. Results are displayed separately for small (≤200 mm^3^) and large (>200 mm^3^) nodules. The black line represents the difference in volume, while the grey line represents the attenuation. The dotted line through the y-axis represents the reference line where there is no difference compared to the volume measured on the conventional reconstruction. Note that the range of the y-axis might differ between the figures. *keV kiloelectron Volt*

In Fig. 1 an example of the image appearance of a particular nodule is provided at the different reconstructions. Table 1 shows absolute volume differences in mm^3^ and relative volume differences in percentages of VNC reconstructions and reconstructions at different keV levels compared to conventional reconstructions. Overall, VNC resulted in 5.5% (2.6 – 11.2%; *p*<0.001) lower nodule volumes compared to conventional reconstructions. The attenuation of the nodules at conventional reconstructions was 49 (17 – 63) HU and -5 (-49 – 12) HU at VNC reconstructions. Forty keV reconstructions resulted in higher volumes (11.2% increase (6.9 – 20.9%)), while volumes at 70 keV reconstructions were comparable to volumes at conventional reconstructions. Higher keV levels resulted in smaller volumes down to -6.3% (-11.0 – -2.2%) at 200 keV. Results for small and large nodules were similar. Fig. 2 shows the relationship between differences in nodule volumes at different mono-energetic levels and the attenuation of nodules. For both small and large nodules, the decrease in volume at higher mono-energetic levels was accompanied by decreased attenuation of nodules. The median Spearman rank correlation was 0.77 (0.49 – 0.94). The correlation was 0.81 (0.49 – 0.91) for small nodules and 0.66 (0.49 – 0.94) for large nodules.

**Table 1:** Difference in volume between conventional reconstructions and VNC and mono-energetic reconstructions

	Absolute difference (mm^3^)	Relative difference (%)	*p*-value
All nodules			
Conventional vs VNC	-9.6 [-24.6 – -3.4]	-5.5 [-11.2 – -2.6]	*p*<0.001
Conventional vs 40 keV	20.1 [8.5 – 43.8]	11.2 [6.9 – 20.9]	*p*<0.001
Conventional vs 70 keV	0.1 [-4.4 – 9.5]	0.1 [-4.2 – 2.9]	*p*=0.540
Conventional vs 100 keV	-6.7 [-14.7 – -1.5]	-3.7 [-7.3 – -1.2]	*p*<0.001
Conventional vs 130 keV	-6.7 [-17.9 – -2.4]	-4.8 [-8.9 – -1.6]	*p*<0.001
Conventional vs 160 keV	-9.5 [-21.2 – -4.0]	-5.6 [-9.1 – -2.7]	*p*<0.001
Conventional vs 200 keV	-8.8 [-22.8 – -3.1]	-6.3 [-11.0 – -2.2]	*p*<0.001
Nodules ≤200mm^3^			
Conventional vs VNC	-7.2 [-13.4 – -3.1]	-9.0 [-13.5 – -2.9]	*p*<0.001
Conventional vs 40 keV	12.4 [7.8 – 25.0]	12.8 [7.6 – 21.7]	*p*<0.001
Conventional vs 70 keV	-0.2 [-3.4 – 3.8]	-0.2 [-4.2 – 2.4]	*p*=0.904
Conventional vs 100 keV	-4.3 [-8.5 – -1.5]	-4.8 [-7.6 – -1.6]	*p*<0.001
Conventional vs 130 keV	-6.2 [-9.7 – -2.6]	-5.2 [-9.2 – -2.3]	*p*<0.001
Conventional vs 160 keV	-6.8 [-11.9 – -3.3]	-6.5 [-10.5 – -3.5]	*p*<0.001
Conventional vs 200 keV	-6.9 [-12.4 – -2.4]	-7.3 [-11.4 – -2.5]	*p*<0.001
Nodules >200mm^3^			
Conventional vs VNC	-26.8 [-79.7 – -6.4]	-4.0 [-8.0 – -1.3]	*p*<0.001
Conventional vs 40 keV	52.4 [24.8 – 120.1]	10.8 [3.5 – 14.1]	*p*<0.001
Conventional vs 70 keV	1.7 [-27.3 – 20.5]	0.7 [-3.6 – 3.0]	*p*=0.757
Conventional vs 100 keV	-14.2 [-57.5 – 0.7]	-2.7 [-4.7 – 0.2]	*p*=0.007
Conventional vs 130 keV	-17.9 [-62.3 – 0.4]	-3.4 [-7.6 – 0.1]	*p*=0.004
Conventional vs 160 keV	-21.6 [-69.5 – -6.7]	-3.8 [-6.7 – -2.3]	*p*<0.001
Conventional vs 200 keV	-41.4 [-65.2 – -4.9]	-4.0 [-9.0 – -1.3]	*p*=0.005

Negative differences mean that the nodules were larger on conventional reconstructions. Bonferroni corrected *p*-values below 0.007 were considered significant *VNC virtual non-contrast, keV kiloelectron Volt.*

**Fig. 3:**
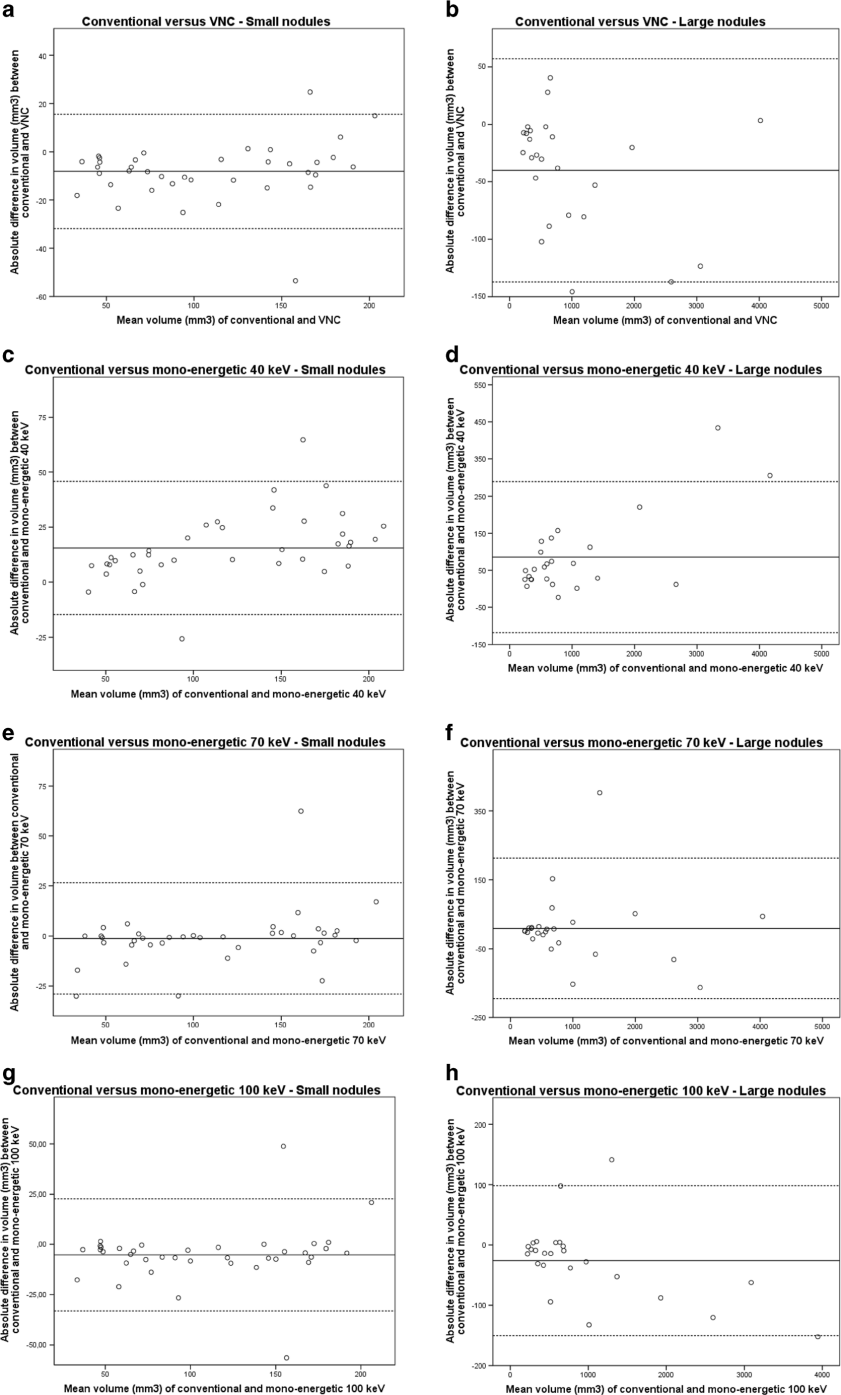

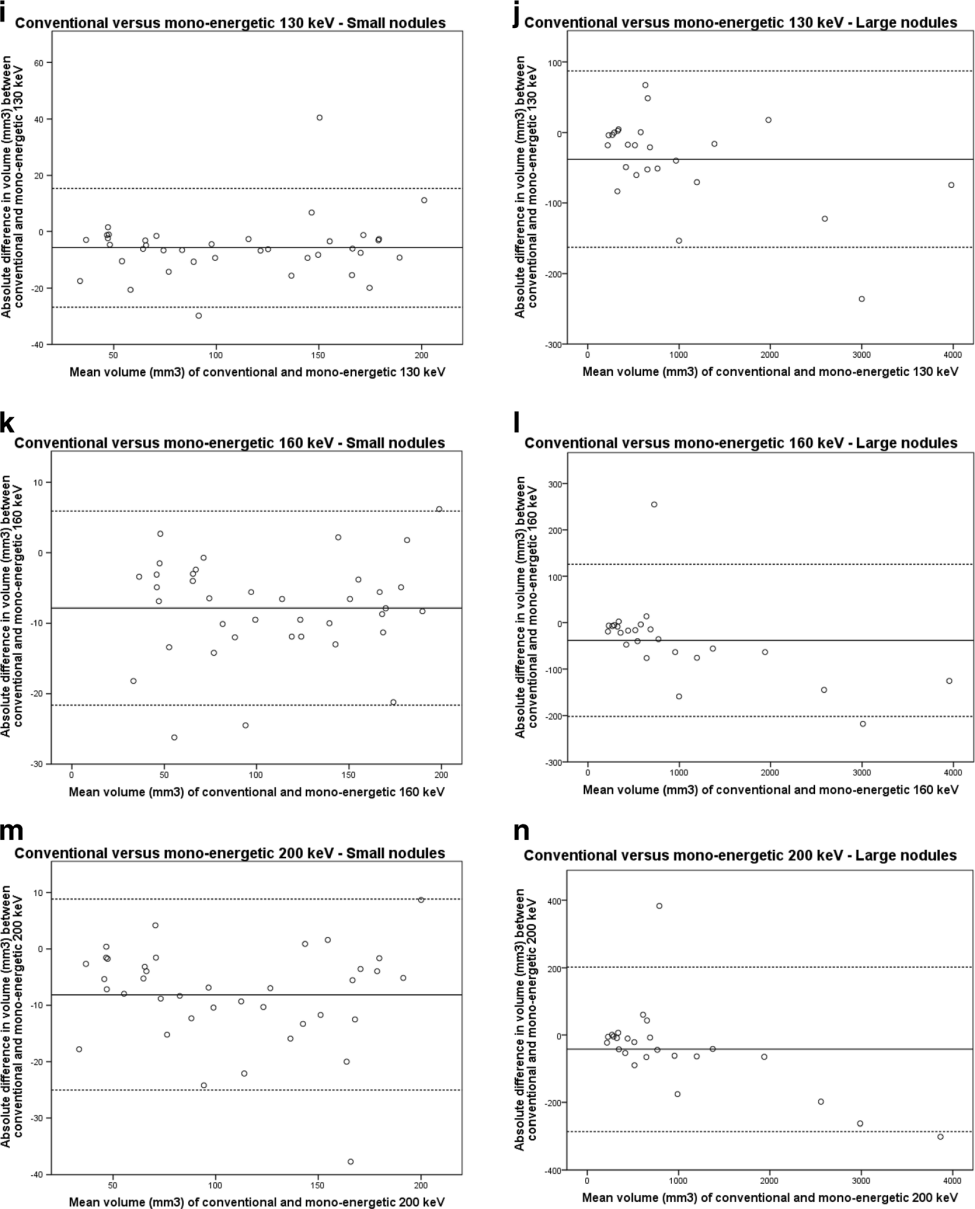
Bland-Altman plots for differences between conventional reconstructions and VNC and mono-energetic reconstructions. The continuous line represents the mean volume difference (mm^3^) while the dotted lines represent the upper and lower limits of agreement. Small nodules were ≤200mm^3^ while large nodules were >200 mm^3^ *. VNC virtual non-contrast, keV kiloelectron Volt*

Bland-Altman plots are provided in Fig. 3 and did not show any systematic bias.

## Discussion

This study shows a strong to excellent correlation between volume and attenuation of pulmonary nodules. VNC and high keV mono-energetic images resulted in low attenuations and low nodule volumes, while low keV mono-energetic images resulted in higher attenuations and nodule volumes. This is probably caused by differences in peripheral enhancement of nodules at different mono-energetic levels, and may also explain the difference in volume between contrast-enhanced and non-contrast-enhanced acquisitions.

Several studies have shown the influence of contrast material on pulmonary nodule volumetry using conventional CT [4, 5, 9, 15, 16]. The largest study was performed by de Jong et al. [9], in which the effect of contrast was investigated in 101 nodules. Patients received both a contrast-enhanced and a low-dose non-contrast enhanced CT, and nodule volumes were compared. Volumes decreased by almost 10% on non-contrast enhanced CT, and this decrease was most evident in small nodules (≤200 mm^3^). Several smaller studies confirmed those findings and described nodule volume decreases of 8% [4], 5% [15] and 3% [16], respectively. However, in all those studies several acquisitions were performed to obtain both a contrast-enhanced and a non-contrast-enhanced acquisition, thereby introducing inter-scan variation, which can lead to differences up to 24% [9, 17]. The differences in those studies can, therefore, also be partly explained by inter-scan variation. Although in the study of the Jong et al. [9] the effect of inter-scan variation was assessed as well, and it was reported to be only 1%. In the current study, the effect of contrast was investigated using a single acquisition, which excludes inter-scan variation as a cause of difference in volume. A systematic underestimation in nodule volume of 5.5% (2.6 – 11.2%) was found for the VNC reconstructions. This is within the range described in previous studies, and shows that this cannot be attributed to inter-scan variation. Furthermore, the mono-energetic reconstructions showed a strong to excellent correlation between volume and attenuation of nodules, which may explain the differences found between contrast and non-contrast acquisitions. It is possible that peripheral enhancement of lung nodules by iodine uptake leads to an enlargement and, thus, overestimation of measured nodule volumes [4]. In contrast-enhanced acquisitions, the difference in HU-value between lung parenchyma and nodules increases, leading to different segmentation margins [15, 16]. In our study, the correlation was stronger for small nodules (Spearman *R*=0.81 (0.49 – 0.91)) compared to larger nodules (Spearman *R*=0.66 (0.49 – 0.94)). In large nodules the surface to volume ratio is smaller compared to small nodules. Since the segmentation margins depend on the contour detection, it is possible that volume measurements of large nodules are less sensitive to attenuation variation compared to small nodules.

Virtual mono-energetic images using dual-energy CT can be reconstructed at any desired energy level between 40 and 200 keV. We found a correlation between the attenuation and the changes in volume. No differences were seen between volumes on conventional reconstructions compared to volumes on reconstructions at 70 keV. This is because the HU values derived at 70 keV are comparable to the HU values derived with conventional images at 120 kVp [13].

Dual-energy CT might also offer other advantages for the evaluation of pulmonary nodules. Differences in enhancement of nodules before and after contrast injection may be an indicator of malignancy and can be assessed with a single contrast-enhanced acquisition using the VNC reconstruction instead of an additional non-contrast enhanced acquisition [18]. Furthermore, calcium can be differentiated from enhanced tissue [12, 19]. Three studies investigated the value of spectral curves and iodine concentrations for the differentiation between benign and malignant nodules, and reported that this could significantly improve the diagnostic accuracy compared to the difference in enhancement between contrast-enhanced and non-contrast-enhanced acquisitions [20–22]. Xiao et al. [20] investigated the difference in iodine concentration between an arterial and venous phase, and reported a significantly higher difference for malignant nodules. This suggests that malignant nodules are better perfused or have a faster washout of contrast.

It is important to note that dual-energy CT can be used without an increased radiation dose. In the current study, the CTDIvol of the acquisition protocol was the same as for conventional CT. Dual-energy potentially offers the opportunity to reduce the radiation dose by eliminating non-contrast acquisitions from multiphase studies and by preventing additional examinations due to improved lesion characterisation [23].

This study has several limitations. First, for most nodules there was no diagnosis based on histopathological biopsy; therefore, no differentiation could be made between malignant and benign nodules. Since malignant nodules seem to enhance more prominently than benign nodules, the correlation between nodule volume and attenuation may be influenced [24]. Second, only a contrast-enhanced acquisition was performed; therefore, the results of the VNC reconstruction could not be compared to a true non-contrast enhanced acquisition. Third, the hardware and software of only one vendor was used and results may differ for other CT systems and software packages.

In conclusion, VNC and mono-energetic images acquired on a novel dual-layer spectral CT system show that higher pulmonary nodule attenuation results in larger nodule volumes. This may explain the reported underestimation in nodule volume on non-contrast enhanced compared to contrast-enhanced acquisitions. The underestimation in pulmonary nodule volume with VNC was comparable to previously reported reductions in volume between contrast and non-contrast-enhanced acquisitions.

## Abbreviations

CT: Computed Tomography
CTDIvol: Volumetric CT Dose Index
DLP: Dose Length Product
HU: Hounsfield Unit
keV: kiloelectron Volt
SSDE: Size-Specific Dose Estimate
VNC: Virtual Non-Contrast
